# Field trial: disinfection of contaminated anesthetic masks for piglets

**DOI:** 10.1186/s40813-023-00321-1

**Published:** 2023-05-26

**Authors:** L. Friedrich, E. Winner, H. Härtel, S. Gumbert, S. Zöls, M. Ritzmann, M. Beisl, K. Kempf, A. von Altrock, N. Kemper, J. Schulz

**Affiliations:** 1 Institute for Animal Hygiene, Animal Welfare and Farm Animal Behaviour, University of Veterinary Medicine Hannover, Foundation, Bischofsholer Damm 15, 30173 Hannover, Germany; 2 Clinic for Swine at the Centre for Clinical Veterinary Medicine, LMU Munich, Sonnenstrasse 16, 85764 Oberschleissheim, Germany; 3 Clinic for Swine, Small Ruminants, Forensic Medicine and Ambulatory Service, University of Veterinary Medicine Hannover, Foundation, Bischofsholer Damm 15, 30173 Hannover, Germany

**Keywords:** Isoflurane, Anesthesia, Anesthetic masks, Castration, Suckling piglets, Hygiene, Disinfection

## Abstract

This paper aimed to assess the success of cleaning and disinfection on microbiological contamination of anesthetic masks, which were used for automated isoflurane anesthesia for surgical castration of male piglets. Data collection took place on 11 farms in Southern Germany between September 2020 and June 2022. Each farm was visited three times (one farm having two different anesthesia devices was visited six times), and microbiological assessments took place at four sample points (SP): after unpacking the masks (SP0), after disinfection before anesthesia (SP1), after anesthesia of all piglets to be castrated in this run (SP2), and after disinfection after anesthesia (SP3). The microbiological assessment included the determination of total bacteria count, total count of hemolytic and non-hemolytic mesophilic aerotolerant bacteria and a qualitative detection of indicator bacteria *Escherichia (E.) coli*, extended-spectrum beta-lactamase-producing *E. coli* (ESBL) and methicillin-resistant *Staphylococcus aureus* (MRSA). For analysis, a generalized linear mixed model was applied using farms and farm visits as random effects and sampling points nested in farm visits as fixed effect. The fixed effect was highly significant for all three variables (total bacteria count, total count of hemolytic and non-hemolytic mesophilic aerotolerant bacteria) (p < 0.001). The bacterial counts at SP0 were about the same as at SP3. Concerning indicator bacteria, their presence was highest at SP2 and lowest at SP3. No indicator bacteria were present at SP1. It can be concluded that disinfection of anesthetic masks, especially before performing anesthesia, may effectively protect piglets of the following batch against unwanted transmission of pathogens. These findings will help farmers plan cleaning and disinfection activities.

## Introduction

As a result of an amendment to the German Animal Welfare Act in 2013 and a 2-year extension of the deadline, the former surgical castration without anesthesia of male suckling piglets less than 8 days old has been prohibited in Germany since 2021 [2]. In Germany, anesthesia for surgical castration in pigs can be applied via injection using azaperone and ketamine and via inhalation using isoflurane [26]. While a veterinarian is required for the injection of anesthesia, isoflurane anesthesia may also be carried out by competent persons, i. e., trained farmers [3]. Currently, Switzerland, where surgical castration of piglets without anesthesia was banned in 2010, is the only country in Europe commonly applying isoflurane for surgical castration of male suckling piglets [6, 7]. Although isoflurane anesthesia is not yet widely used in livestock farming, inhalation anesthesia is of general importance for veterinary surgery, especially concerning small animals. In pigs, and basically also in small animals, using anesthetic masks results in a potential risk of unwanted transmission of pathogens, zoonotic agents and resistant bacteria from animal to animal or from animal groups to other animal groups, respectively. For instance, piglets may carry infectious agents or resistant zoonotic bacteria in the nose, mucus, and planum rostrale [22, 23]. Mucus and epithelia come into contact with the inner surfaces of anesthetic masks and thereby may contaminate them. Hitherto, only a few data have been published about the contamination of anesthetic masks for piglets and the effect of measures to avoid the transmission of microbial contaminations, e. g. Weber et al. [27]. In the present study, automated isoflurane anesthesia is applied for the surgical castration of male suckling piglets. The present paper aims to assess the microbiological assessment of the success of cleaning and disinfection of used anesthetic masks to reduce the potential risk of unwanted pathogen transmission from animal to animal. These findings will help farmers plan cleaning and disinfection activities and provide a principal approach concerning the hygiene management using inhalation anesthesia.

## Material and methods

### Data collection

The data collection occurred between September 2020 and June 2022 on 11 farrowing farms. The farms were selected with the help of several veterinary practitioners, and participation in the study was voluntary for the farms. Due to the repeated sampling, primarily farms in Southern Germany were included in the study. In order to obtain a representative sample, the farms differed in farm size and production rhythm (Table 1).

**Table 1:** Overview of the farms (see also [28])

Farm^1^	Anesthesia device^2^	The average number of sows, farrowing rhythm in weeks, piglets/batch and routine management procedures on castration day
2	PN	100–250, 3 wks, 102, ear tag
6	PN	100–250, 3 wks, 101
1	PA	100–250, 3 wks, 101, iron
3	PA	> 250, 1–1-0, 74, tail dock
4	PA	100–250, 3 wks, 82, iron
5	PA	< 100, 3 wks, 47
7	AN	> 250, 3 wks, 371
8	AN	100–250, 3 wks, 86, ear tag
9	AN	100–250, 3 wks, 52, tail dock, iron
10	AN	< 100, 3 wks, 48, ear tag
13	PSn	> 250, 3 wks, 243, ear tag, (iron)
3	PSl	> 250, 1–1-0, 74, tail dock
11 farms (one farm with two different devices)	5 devices	

^1^Corresponding to [28]

^2^PN: PigNap 4.0, PA: PorcAnest 3000, AN: Anestacia, Psn: PigletSnoozer, Psl: PigletSleeper

On these farms, male suckling piglets less than 8 days old were surgically castrated under isoflurane anesthesia by the farmers according to the German Animal Welfare Act and corresponding regulations [3]. Five different anesthesia devices were used in the present study: PigNap 4.0 (PN), Anestacia (AN), PorcAnest 3000 (PA), PigletSnoozer (PSn), and PigSleeper (PSl). The devices differed in various characteristics. The characteristics of PN, AN, and PA are described in Winner et al. [28]. Videos of all devices are available on the internet: https://www.landwirtschaftskammer.de/landwirtschaft/tiergesundheit/sgd/isofluran-videos.htm.

**Fig. 1:**
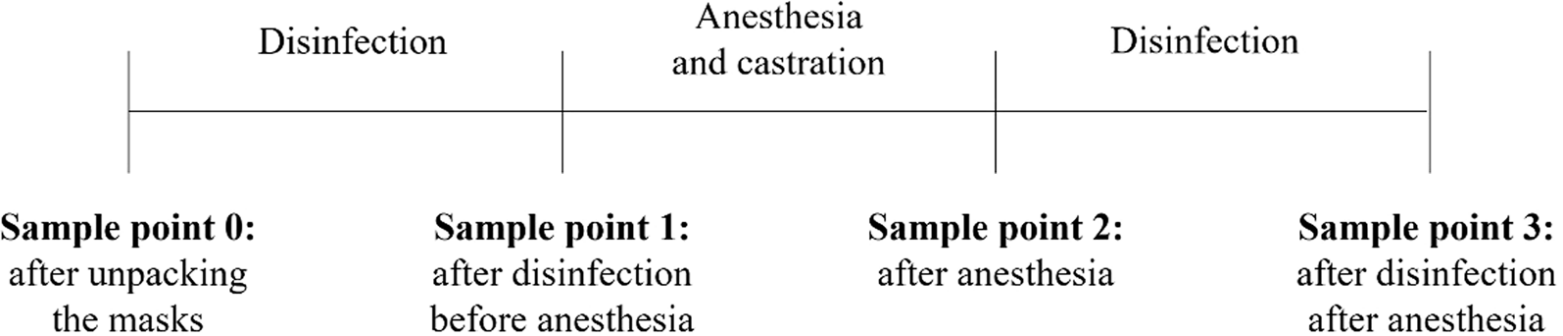
Graphical illustration of the workflow of the present study and corresponding sample points.

For the present study, each farm was visited three times. On farm number 3 (Table 1), two different devices were used. Thus, this farm was visited six times in total. Before the first sampling at the first visit, all masks were wiped with alcohol as a baseline. In general, in each farm visit, a microbiological assessment of the anesthetic masks took place at four different sample points: after unpacking the masks (sample point 0), after disinfection before anesthesia (sample point 1), after anesthesia (sample point 2), and after disinfection after anesthesia (sample point 3) (Fig. 1). For feasibility reasons, samplings (and corresponding cleaning or disinfection) were performed at each sample point after all piglets passed the sample point (Ø 115 (± 104) piglets per batch, min: 25, max: 469). Each farm visit comprised only one batch. Thus, in total, each farm visit resulted in four samples.

For cleaning, the masks were cleaned with tap water and dried with disposable paper towels. For disinfection, the disinfectant available on the farm was used. In most farms, the disinfection was conducted with Meliseptol^®^ New Formula (B. Braun SE, Germany), a ready-to-use alcohol-based disinfectant for spraying on or wiping off. Farms 4 and 10 used Kodan^®^ Tinktur Forte (Schülke, Germany), a disinfectant containing 2-Propanol, 1-Propanol, and Biphenyl-2-ol. The disinfectant was not changed during the data collection. The masks were wetted with the disinfectant, allowed to dry for a short time and then wiped dry with a sterile compress. After disinfection after anesthesia, the masks were packed in ziploc bags and stored in the barn until the next data collection (sample point 0) (time stored Ø 102 (± 113) days, min: 8, max: 430).

For the microbiological assessment, the masks’ inner surfaces were first sampled using a sterile swab soaked with 0.9% NaCl solution and then with a second sterile dry swab at each of the four sample points. Four swabs were used for the mask of the PN, as it is designed with a double wall; one dry and one soaked swab each for the inner and outer mask. The samplings were always carried out by the same two pre-trained veterinarians from the Clinic for Swine at the Centre for Clinical Veterinary Medicine, LMU Munich, Germany. The two or four swabs per sample were transferred to a single tube with 10 ml phosphate buffer saline (PBS) and 0.01% Tween 20 for subsequent analyses and brought to the laboratory under cooled conditions. As transport control, one tube with 0.9% NaCl solution and one tube with PBS and 0.01% Tween 20 were enclosed. All samples were sent overnight and were processed in the laboratory within 24 h. Upon arrival at the laboratory, the swab samples were vortexed in the tubes for 1 min at maximum speed (uniTexer1, LLG Labware, Meckenheim, Germany). The microbiological assessment included the determination of the total bacteria count, the total count of hemolytic and non-hemolytic mesophilic aerotolerant bacteria and the qualitative detection of the indicator bacteria *Escherichia (E.) coli*, extended-spectrum beta-lactamase-producing *E. coli* (ESBL) and methicillin-resistant *Staphylococcus aureus* (MRSA).

To determine the total bacteria count and the total count of mesophilic aerotolerant bacteria, serial dilutions were prepared with 0.9% NaCl solution and 0.1 ml was plated out on three blood-based agar plates (VWR, 84,647.0500) and three azide blood agar plates (Oxoid, CM0259) with sheep blood (Oxoid, SR0051C), respectively. Both plates were incubated at 36 °C, the azide blood agar plates with 5% carbon dioxide (CO_2_). After 24 h of incubation for the total bacteria count and 48 h of incubation for both, colony-forming units (CFU) were counted, and the results were expressed in CFU/mask by extrapolating the serial dilutions factor to the distributed volume. Concerning mesophilic aerotolerant bacteria it was distinguished between hemolytic and non-hemolytic bacteria. Isolates from pig barns cultivated on azide blood agar and incubated at 5% CO_2_ typically belong to the order of *Lactobacillales* and to genera that form cocci [17]. Therefore, counts of these bacteria were hereafter considered hemolytic and non-hemolytic cocci. Qualitatively, the presence of *E. coli*, ESBL and MRSA was determined after pre-enrichment and as described by Friese et al. [8] and Ahmed et al. [1].

The presence of *E. coli*, ESBL and MRSA was confirmed by MALDI-TOF mass spectral analysis. The suspected MRSA isolates were confirmed by real-time PCR (QuickBlue Realquick QB-RTi-39, Q-Bioanalytic GmbH, Bremerhaven, Germany).

### Statistical analysis

Data processing and statistical analyses were performed using SAS 9.4 [16]. First, the analysis compared the total bacteria count per mask, the total count of non-hemolytic cocci per mask and the total count of hemolytic cocci per mask between the different sample points. Were the CFU of the variables was 0, these were replaced by 0.5, according to Daly und Harris [5], to allow for log transformation. Subsequently, a generalized linear mixed model was applied using farms and farm visits as random effects and sampling points nested in farm visits as fixed effect to evaluate whether there was a significant difference between the sample points. No discrimination was made between farms or types of masks. Results with a p-value < 0.05 were considered statistically significant. If significant differences were found between more than two sample points, pairwise comparisons between sample points using differences of least squares means and Bonferroni adjustment were conducted. Since sample point 0 occurred only in visits 2 and 3 (to evaluate disinfection and packaging after use, the masks had to be used first for anesthesia), the first visit on each farm was excluded from the analyses. Thus, the data set to be analyzed included 11 farms (on farm number 3, two different devices were used, thus, this farm appears twice in the analysis), each with two visits and four sample points.

Second, the occurrence of the indicator bacteria *E. coli*, ESBL and MRSA was evaluated using descriptive statistics. For this purpose, the indicator bacteria detection frequency at the respective sample points was compared.

## Results

**Fig. 2:**
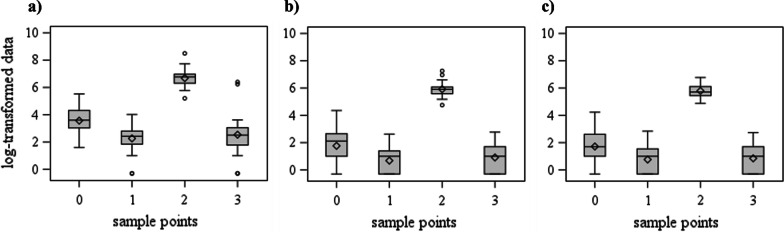
Log-transformed data of **a** total bacteria count per mask, **b** total count of non-hemolytic cocci per mask, and **c** total count of hemolytic cocci per mask per sample point (0: after unpacking the masks, 1: after disinfection before anesthesia, 2: after anesthesia, 3: after disinfection after anesthesia, over all farms).

The log-transformed data of total bacteria count per mask, of total count of non-hemolytic cocci per mask, and of total count of hemolytic cocci per mask per sample point over all farms are shown in Fig. 2.

All three variables show a concurrent pattern with a higher total count after anesthesia (sample point 2) and the lowest total count after disinfection before anesthesia (sample point 1). Besides, the total bacteria count per mask is two log levels higher than the total count of cocci, both hemolytic and non-hemolytic. After cleaning and disinfection of used masks, the total bacteria and cocci reduction is approximately on the same level (sample point 3).

The results of the generalized linear mixed model are shown in Table 2.

**Table 2:** Results of the analysis of variance with repeated measurements

Variable: fixed effect	Level^1^	Mean	95% confidence interval	p-value
Total bacteria count per mask: sample point				< 0.001
	0	3.57	3.13;4.00	
	1	1.96	1.50;2.42	
	2	6.68	6.42;6.93	
	3	2.48	2.06;2.89	
Total count of non-hemolytic cocci per mask: sample point				< 0.001
	0	1.80	1.27;2.34	
	1	0.22	− 0.34;0.78	
	2	5.88	5.63;6.13	
	3	0.74	0.22;1.26	
Total count of hemolytic cocci per mask: sample point				< 0.001
	0	1.76	1.24;2.27	
	1	0.63	0.15;1.11	
	2	5.79	5.55;6.03	
	3	0.78	0.22;1.33	

^1^0: after unpacking the masks, 1: after disinfection before anesthesia, 2: after anesthesia, 3: after disinfection after anesthesia

The fixed effect sample point was highly significant for all three variables (p < 0.001). The bacterial counts after disinfection and before the use of the masks were at about the same level as after disinfection after anesthesia (sample points 1 and 3).

Table 3 contains the differences between the least-square means of the pairwise comparisons for the fixed effect.

**Table 3:** Differences between least-square means of the significant fixed effect sample point. P-values were adjusted by applying post hoc Bonferroni adjustment

First sample point under comparison^1^	Second sample point under comparison^1^	Total bacteria count per mask		Total count of non-hemolytic cocci per mask		Total count of hemolytic cocci per mask	
		Estimate	p-value	Estimate	p-value	Estimate	p-value
0	1	1.61	< 0.001	1.58	< 0.001	1.13	< 0.001
0	2	-3.22	< 0.001	-4.08	< 0.001	− 4.03	< 0.001
0	3	1.16	< 0.001	1.06	< 0.01	0.98	< 0.01
1	2	4.83	< 0.001	5.66	< 0.001	5.16	< 0.001
1	3	0.45	0.15	0.52	0.63	0.14	1.00
2	3	4.38	< 0.001	5.14	< 0.001	5.02	< 0.001

^1^0: after unpacking the masks, 1: after disinfection before anesthesia, 2: after anesthesia, 3: after disinfection after anesthesia

In all three variables, the pairwise comparisons revealed only non-significant differences between sample points 1 and 3, which are not consecutive sample points, either way. The differences were significant in all other pairwise comparisons, especially the consecutive comparisons (p < 0.05). Within the consecutive sample points, the differences were highest for comparing sample points 1 and 2 (after disinfection before anesthesia vs. after anesthesia) and 2 and 3 (after anesthesia vs. after disinfection after anesthesia). Hence, disinfection after anesthesia reduced both the total bacterial count and the total count of non-hemolytic and hemolytic cocci by around 5 logs.

Concerning the indicator bacteria, the presence of indicator bacteria *E. coli*, ESBL and MRSA was highest after anesthesia (sample point 2) and lowered after disinfection (sample point 3). No indicator bacteria were present after disinfection before anesthesia (sample point 1) (Table 4).

**Table 4:** Frequency of samples [%] with indicator bacteria (*Escherichia (E.) coli*, extended-spectrum beta-lactamase-producing *E. coli* (ESBL) and methicillin-resistant *Staphylococcus aureus* (MRSA) at the respective sample points (0–3)

Indicator bacteria	0: After unpacking the masks	1: After disinfection before anesthesia	2: After anesthesia	3: After disinfection after anesthesia
*E. coli*	8.33	0.00	87.5	4.17
ESBL	0.00	0.00	12.5	4.17
MRSA	8.33	0.00	66.7	4.17

## Discussion

The study was conducted on 11 farms in Southern Germany. Their participation in this study was voluntary. Thus, this study has to be classified as a field trial and its results need to be further validated before they can be presented as being generally applicable. However, including farms with different characteristics, it was ensured that the results are of general importance for on-farm application.

The results of the present study indicate that piglets’ snouts microbiologically contaminated anesthetic masks during anesthesia. This was also shown in a preliminary study by Härtel et al. [9]. The microbiological contamination of anesthetic masks during anesthesia can present a potential risk of spreading pathogens, zoonotic agents and resistant bacteria. This matters not only for livestock production, but is also important for inhalation anesthesia in small animal surgery.

To depict the microbial reduction due to hygiene measures, the microbiological assessments included determining the total bacteria count and the total count of hemolytic and non-hemolytic cocci. The total bacteria count and the count of aerotolerant cocci were used because an earlier study showed that these parameters were useful for assessing a significant quantitative reduction by a disinfection measure in pig farms [17]. *E coli*, ESBL, and MRSA served as indicator bacteria because they represent potential pathogenic, zoonotic, and resistant bacteria that could be expected on pig farms. More details are illustrated subsequently:

Considering van Beers‐Schreurs et al. [22], *E. coli* is part of the pigs’ normal flora. However, pathogenic *E. coli* causes colibacillosis, one of the swine industry’s most significant diseases. Over the past decades, resistant and multiresistant isolates have emerged [18]. Concerning the resistance to cephalosporins, the major mechanism relies on the production of ESBL [14]. This is a major challenge in controlling piglet diarrhea in swine production. However, neither the pathogenicity of *E. coli* and ESBL isolates nor potential resistance mechanisms were analyzed in the present study. As used in our study, no relevant changes in minimum inhibitory concentration (MIC) values have yet been reported for disinfectants containing alcohols [15]. Therefore, the effective disinfection of the chosen indicator bacteria suggests the same effectivity against pathogenic strains.

Besides diarrheal pathogens, respiratory pathogens are particularly relevant in pig production. Respiratory diseases are also referred to as respiratory disease complex (PRDC) since its etiology is multifactorial, caused by a variety of bacteria and viruses [10, 20]. Although the disinfectant efficacy against viruses was not investigated in the present study, Turner and Burton [21] showed in a review that 70% ethanol has been found in many studies to be highly effective in inactivating viruses in pig slurry. Therefore, it can be expected that the disinfection method tested in the present study is also useful to prevent from respiratory diseases.

Alcoholic disinfectants act, in general, against a broad spectrum of bacteria and viruses, but in cases of known problems with pathogens (bacterial spores, some viruses) that cannot be efficiently inactivated, it can be recommended to adapt the disinfectant. In this context, potential skin irritations at the snout of piglets and the toxicity of disinfectants should be considered. Notwithstanding this, the disinfection of cleaned masks before anesthesia (sample point 1) revealed no detection of indicator bacteria although sensitive enrichment methods were used, which increased the likelihood of detection [1]. In summary, if indicator bacteria are eliminated, the question of pathogenicity and resistance mechanisms no longer arises.

Concerning resistant bacteria, in 2005, a new methicillin-resistant *Staphylococcus aureus* (MRSA), now called livestock-associated (LA)-MRSA, was found on pig farms in the Netherlands [25]. Even though many pigs carry LA-MRSA, infections are unusual [23]. Colonized pigs, however, can serve as MRSA reservoirs for the human population [4, 19]. This is why it is important to reduce or prevent animal colonization [24]. As shown for *E. coli* and ESBL, MRSA was efficiently eliminated by the second disinfection of the cleaned mask immediately before anesthesia (sample point 1) (Table 4).

Despite these explanations, it must be considered that transmission of pathogens occurs in groups of animals that have contact with each other or are housed in the same barn [11, 12]. Considering feasibility, it is more convenient to clean and disinfect the masks after each batch (as conducted in the present study) than after each anesthetized piglet. The results of the present study suggest that this approach seems to allow interrupting chains of infection between different animal groups. This is also shown by the fact that the reduction in bacteria was generally high, even though small and large batches were analyzed together. Further, no discrimination was made between farms or types of masks within the present study since the cleaning and disinfection measures have to work on every farm and every type of mask to make a recommendation that is generally applicable.

The results show that disinfection after anesthesia (sample point 2) reduced both the total bacteria count and the total count of non-hemolytic and hemolytic cocci by around 5 logs, regardless of the anesthesia device/mask used. Swab sampling followed by dilution of the washing buffer are suitable methods to quantify the bacterial loads from surfaces in animal houses [13]. Although the applied cultivation procedures might not have detected all cultivable bacteria from the inner surface of the masks, for instance, slow-growing bacteria, the quantification of both total bacteria and non-hemolytic and hemolytic cocci showed the same trends (Fig. 2) and highly significant reductions after disinfection (Table 3). Further, disinfection after anesthesia (sample point 2) lowered the presence of indicator bacteria. The lowest total bacteria count and the lowest total count of non-hemolytic and hemolytic cocci, as well as zero presence of indicator bacteria, could be detected when prior cleaned and disinfected masks were unpacked and disinfected before anesthesia (sample point 1). This could suggest that disinfection before anesthesia is, in general, used. However, a low level of bacteria was found even after unpacking cleaned and disinfected masks (sample point 0). Although disinfection after anesthesia and packaging may lead to a successful interruption of infection chains, a second disinfection immediately before anesthesia can be recommended on farms and would be feasible.

## Conclusion

Disinfection of an anesthetic mask is important since it may prevent unwanted transmission of pathogens, zoonotic agents, and resistant bacteria between animal groups. The results of the present study confirm that disinfection of anesthetic masks effectively reduces the total bacteria count and even eliminates indicator bacteria. A significant reduction of bacteria by mask hygiene between the use in different batches is, therefore, a successful preventive measure in the sense of interrupting infection chains and unwanted colonization and can be recommended to the farmers.

## Data Availability

The datasets generated and analyzed during the current study are not publicly available since the study has not been completed yet but the data are available from the corresponding author on reasonable request.

## References

[1] Ahmed MFE, Ramadan H, Seinige D, Kehrenberg C, El-Wahab A, Volkmann N, Kemper N, Schulz J (2020). Occurrence of extended-spectrum beta-lactamase-producing Enterobacteriaceae, microbial loads, and endotoxin levels in dust from laying hen houses in Egypt. BMC Vet Res.

[2] Anonymous, 2018. Entwurf eines Vierten Gesetzes zur Änderung des Tierschutzgesetzes: Drucksache 19/5522. Online: https://dserver.bundestag.de/btd/19/055/1905522.pdf.

[3] Anonymous. Verordnung zur Durchführung der Betäubung mit Isofluran bei der Ferkelkastration durch sachkundige Personen (Ferkelbetäubungssachkundeverordnung, FerkBetSachkV); 2020.

[4] Cuny C, Nathaus R, Layer F, Strommenger B, Altmann D, Witte W (2009). Nasal colonization of humans with methicillin-resistant Staphylococcus aureus (MRSA) CC398 with and without exposure to pigs. PLoS ONE.

[5] Daly SW, Harris AR (2022). Modeling exposure to fecal contamination in drinking water due to multiple water source use. Environ Sci Technol.

[6] Enz A, Schüpbach-Regula G, Bettschart R, Fuschini E, Bürgi E, Sidler X (2013). Erfahrungen zur Schmerzausschaltung bei der Ferkelkastration in der Schweiz Teil 1: Inhalationsanästhesie. Schweiz Arch Tierheilkd.

[7] Fredriksen B, Furnols MF, Lundström K, Migdal W, Prunier A, Tuyttens F, Bonneau M (2009). Practice on castration of piglets in Europe. Animal.

[8] Friese A, Schulz J, Hoehle L, Fetsch A, Tenhagen B-A, Hartung J, Roesler U (2012). Occurrence of MRSA in air and housing environment of pig barns. Vet Microbiol.

[9] Härtel H, Gumbert S, Rauh A, Beisl M, Schulz J, Kempf K, Senf S, Winner E, Weiß C, Nüßlein A, Zablotski Y, Ritzmann M, Zöls S (2021). Untersuchungen zur automatisierten Isoflurannarkose bei der Saugferkelkastration. Tierarztl Prax Ausg G Grosstiere Nutztiere.

[10] Hansen MS, Pors SE, Jensen HE, Bille-Hansen V, Bisgaard M, Flachs EM, Nielsen OL (2010). An investigation of the pathology and pathogens associated with porcine respiratory disease complex in Denmark. J Comp Pathol.

[11] Maes D, Verdonck M, Deluyker H, de Kruif A (1996). Enzootic pneumonia in pigs. Vet Q.

[12] Marois C, Le Carrou J, Kobisch M, Gautier-Bouchardon AV (2007). Isolation of Mycoplasma hyopneumoniae from different sampling sites in experimentally infected and contact SPF piglets. Vet Microbiol.

[13] Mateus-Vargas RH, Butenholz K, Volkmann N, Sürie C, Kemper N, Schulz J (2022). Boot swabs to evaluate cleaning and disinfection success in Poultry Barns. Agriculture.

[14] Perez F, Endimiani A, Hujer KM, Bonomo RA (2007). The continuing challenge of ESBLs. Curr Opin Pharmacol.

[15] Rozman U, Pušnik M, Kmetec S, Duh D, Šostar Turk S (2021). Reduced susceptibility and increased resistance of bacteria against disinfectants: a systematic review. Microorganisms.

[16] SAS Institute, 2017. Base SAS 9.4 procedures guide: Statistical procedures. SAS Institute.

[17] Schulz J, Bao E, Clauss M, Hartung J (2013). The potential of a new air cleaner to reduce airborne microorganisms in pig house air: preliminary results. Berl Munch Tierarztl Wochenschr.

[18] Seiffert SN, Hilty M, Perreten V, Endimiani A (2013). Extended-spectrum cephalosporin-resistant Gram-negative organisms in livestock: an emerging problem for human health?. Drug Resist Updates.

[19] Smith TC, Pearson N (2011). The emergence of Staphylococcus aureus ST398. Vector-Borne Zoonotic Diseases.

[20] Thacker EL (2001). Immunology of the porcine respiratory disease complex. Vet Clin N Am Food Anim Pract.

[21] Turner C, Burton CH (1997). The inactivation of viruses in pig slurries: a review. Biores Technol.

[22] van Beers-Schreurs HM, Vellenga L, Wensing T, Breukink HJ (1992). The pathogenesis of the post-weaning syndrome in weaned piglets; a review. Vet Q.

[23] van Duijkeren E, Jansen MD, Flemming SC, de Neeling H, Wagenaar JA, Schoormans AHW, van Nes A, Fluit AC (2007). Methicillin-resistant Staphylococcus aureus in pigs with exudative epidermitis. Emerg Infect Dis.

[24] Verhegghe M, Pletinckx LJ, Crombé F, van Weyenberg S, Haesebrouck F, Butaye P, Heyndrickx M, Rasschaert G (2013). Cohort study for the presence of livestock-associated MRSA in piglets: effect of sow status at farrowing and determination of the piglet colonization age. Vet Microbiol.

[25] Voss A, Loeffen F, Bakker J, Klaassen C, Wulf M (2005). Methicillin-resistant Staphylococcus aureus in pig farming. Emerg Infect Dis.

[26] Waldmann KH, Potschka H, Lahrmann KH, Kästner S (2018). Saugferkelkastration unter Lokalanaesthesie. Eine Situationsanalyse aus wissenschaftlicher Sicht. Dtsch Tierärztebl.

[27] Weber S, Daş G, Schulz J, Moors E, Hartung J, Waldmann KH, Gauly M (2013). Isoflurane-anaesthesia used for piglet-castration: a bacteriological assessment of the anaesthetic device. Berl Munch Tierarztl Wochenschr.

[28] Winner E-M, Beisl M, Gumbert S, Härtel H, Kaiser J, Wernecke A, Senf S, Zablotski Y, Ritzmann M, Zöls S (2022). Implementation of piglet castration under inhalation anaesthesia on farrowing farms. Porcine Health Manag.

